# Finding new edges: systems approaches to MTOR signaling

**DOI:** 10.1042/BST20190730

**Published:** 2021-02-05

**Authors:** Alexander Martin Heberle, Ulrike Rehbein, Maria Rodríguez Peiris, Kathrin Thedieck

**Affiliations:** 1 Institute of Biochemistry and Center for Molecular Biosciences Innsbruck, University of Innsbruck, Innsbruck, Austria; 2 Laboratory of Pediatrics, Section Systems Medicine of Metabolism and Signaling, University of Groningen, University Medical Center Groningen, Groningen, The Netherlands; 3 Department for Neuroscience, School of Medicine and Health Sciences, Carl von Ossietzky University Oldenburg, Oldenburg, Germany

**Keywords:** amino acids, computational models, mechanistic target of rapamycin, protein kinase B, signaling, systems biology

## Abstract

Cells have evolved highly intertwined kinase networks to finely tune cellular homeostasis to the environment. The network converging on the mechanistic target of rapamycin (MTOR) kinase constitutes a central hub that integrates metabolic signals and adapts cellular metabolism and functions to nutritional changes and stress. Feedforward and feedback loops, crosstalks and a plethora of modulators finely balance MTOR-driven anabolic and catabolic processes. This complexity renders it difficult — if not impossible — to intuitively decipher signaling dynamics and network topology. Over the last two decades, systems approaches have emerged as powerful tools to simulate signaling network dynamics and responses. In this review, we discuss the contribution of systems studies to the discovery of novel edges and modulators in the MTOR network in healthy cells and in disease.

## Introduction

Kinase signaling networks are a prime example of highly dynamic biological systems whose outputs cannot be fully understood by a static view of their single components. Over the last years, detailed molecular studies of signaling proteins have been increasingly complemented with systems approaches that allow us to understand the dynamic network-tuning arising for instance from interconnected feedback and feedforward loops [1,2]. Fundamental concepts of signal transduction, linked first to biology under the term of *cybernetics* [3,4] and introduced later to cell signaling e.g. by seminal work of Goldbeter [5], Tyson and Novak [6], are now investigated by a growing community of life scientists.

In cell signaling research, systems models informed by time-series data are used to simulate the adaptation of a signaling network to multiple inputs or perturbations, including drug treatments. Such strategies serve for instance to dissect the convergence of known feedforward and feedback loops on a common effector to predict the outcome of a drug perturbation. Furthermore, novel network nodes (e.g. proteins) and connections (e.g. protein–protein interactions) can be postulated and the likelihood of alternative hypotheses can be compared in a quantitative manner. Simulations of signaling outputs arising from alternative network topologies can guide the experimentation to test those hypotheses. Hence, the classical iterative workflow of theoretical and experimental physics is now being translated to the life sciences, and theoretical and experimental biology and medicine work hand in hand.

The tools and methodologies in theoretical biology are as diverse as in the experimental life sciences and they are constantly developing according to the specific biological problems that are being investigated. For instance, theoreticians develop new ways to deal with noisy data [7,8] or non-equidistant dynamic measurements [9–11]. Likewise, experimentalists develop new methods to satisfy the demand for higher quantitative accuracy [12–14] enabling in turn new modeling approaches [12,15–17] relying e.g. on absolute quantitative data. Given the complexity and diversity of the questions that are addressed by systems biology and medicine, there is no single correct approach to a given problem. Yet, conventions arise for certain problems and the call for standardization becomes increasingly urgent to guarantee the quality and reproducibility of the scientific results from theoretical and experimental biology [18–21].

Modeling studies are performed based on prior data, and they generate hypotheses that are tested in subsequent experiments, which in turn can be incorporated into the models. Such iterative combination of *in silico* network modeling with experimental time-series data and validation provides a powerful means to understand the behavior of biological networks in a feasible time frame and work effort. Given the size of the field and multiplicity of problems and studies, we won't attempt a comprehensive overview. Instead, we will outline recent developments and applications focusing on the signaling networks converging on the metabolic master regulator MTOR. We discuss systems approaches of the last decade, which identified and experimentally validated novel edges in the MTOR network, focusing on ordinary differential equation (ODE)-based models constituting the majority of dynamic systems studies on MTOR [1].

## The MTOR signaling network

Cells are living systems, which constantly exchange information with their environment. Environmental inputs are translated into cellular signals that are transmitted through signaling networks to elicit responses that enable a cell to adapt to its environment. The serine/threonine protein kinase MTOR is at the centre of such a network which in response to metabolic signals promotes anabolism and inhibits catabolism [22]. A complex network integrating a multitude of extrinsic and intrinsic cues, intertwined feedback and feedforward mechanisms, and multi-level crosstalk with ancillary signaling networks allows to finely adapt MTOR activity and its downstream processes to the availability of nutrients and to stresses imposed by the environment.

**Figure 1.**
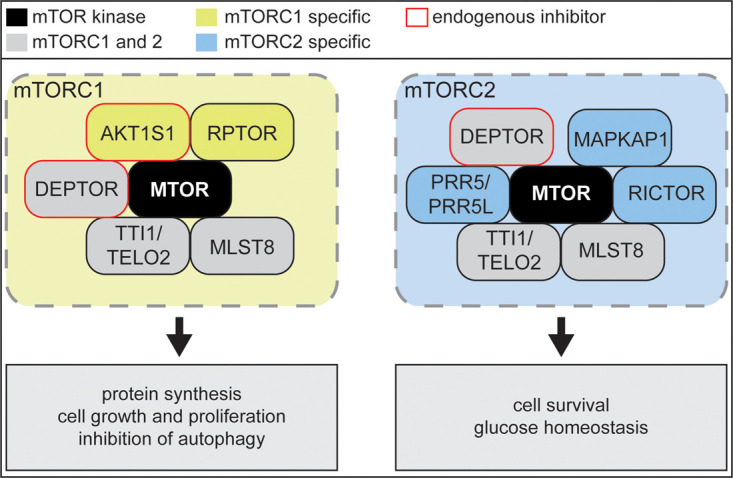
MTOR kinase resides in the two distinct multiprotein complexes mTOR complex 1 (mTORC1, yellow) and mTORC2 (blue). mTORC1 and mTORC2 specific binding partners are shown in yellow or blue, respectively. Shared interactors are shown in grey. Selected processes downstream of the two complexes are depicted at the bottom.

MTOR kinase resides in two distinct multiprotein complexes, termed mTOR complex 1 (mTORC1) and mTORC2 (reviewed by Saxton and Sabatini [23], and Razquin Navas and Thedieck [24]) (Figure 1). mTORC1 comprises the specific binding partner RPTOR (regulatory associated protein of mTORC1) [25,26] and the inhibitory subunit AKT1S1 (AKT1 substrate 1) [27–30], while mTORC2 contains the specific binding partners RICTOR (RPTOR independent companion of mTORC2) [31,32], MAPKAP1 (MAPK associated protein 1) [33,34] and PRR5/PRR5L (Proline rich 5/like) [28,35]. Both complexes share the interactors MLST8 (MTOR associated protein, LST8 homolog) [36], TTI1/TELO2 (TELO2 interacting protein 1/telomere maintenance 2) [37] and the endogenous inhibitor DEPTOR (DEP domain containing MTOR interacting protein) [38]. The two complexes differ not only in structure but also regarding their substrates and localization (reviewed by Betz and Hall [39]) and are embedded in two distinct — yet linked — signaling networks. Hence, mTORC1 and 2 regulate cellular processes in different ways (Figure 1). mTORC1 promotes protein synthesis, while inhibiting autophagy, ultimately enhancing cell growth and proliferation. mTORC2 links to processes such as cell survival and glucose homeostasis [23].

Since the discovery of mTORC1 [25,26] and mTORC2 [31,32] in the early 2000's new modulators and interactions continue to be discovered, forming an ever-growing ramified and multiply-intertwined network. In recent years, *in silico* systems biology approaches have emerged as valuable tools to gain a comprehensive understanding of the topology and dynamic behavior of the MTOR network and identify novel edges by simulating the dynamics of signaling networks converging on mTORC1 and mTORC2.

## Finding new edges in the MTOR network

We discuss in the following the response of the MTOR network to growth factors, amino acids and stressors (reviewed by Liu and Sabatini [22], Razquin Navas and Thedieck [24], Kim and Guan [40], Fu and Hall [41], Heberle et al. [42]), while highlighting molecular edges whose discovery was aided by computational modeling (Table 1).

**Table 1:** Computational studies of the MTOR network

ID	Title	Year	Citation	Experimental treatment	Cell/animal system
a	Insulin Signaling in Type 2 Diabetes: experimental and modeling analyses reveal mechanisms of insulin resistance in human adipocytes	2013	Braennmark et al. [68]	insulin: - steady state, different concentrations - time course	primary human mature adipocytes: healthy and obese individuals with T2D
b	Systems-wide Experimental and Modeling Analysis of Insulin Signaling through Forkhead Box Protein O1 (FOXO1) in Human Adipocytes, Normally and in Type 2 Diabetes	2016	Rajan et al. [69]	insulin: - steady state, different concentrations - time course	primary human mature adipocytes: healthy and obese individuals with T2D
c	Inhibition of FOXO1 transcription factor in primary human adipocytes mimics the insulin-resistant state of type 2 diabetes	2018	Rajan et al. [70]	insulin: - steady state, different concentrations - time course	primary human mature adipocytes human adipose-derived stem cells both expressed dominant negative-FOXO1 or wildtype-FOXO1
d	Crosstalks via mTORC2 can explain enhanced activation in response to insulin in diabetic patients	2017	Magnusson et al. [71]	phosphoproteome data from insulin treated 3T3-L1 adipocytes insulin time course in primary adipocytes	3T3-L1 adipocytes primary human mature adipocytes: healthy and obese individuals with T2D
e	A Single Mechanism Can Explain Network-wide Insulin Resistance in Adipocytes from Obese Patients with Type 2 Diabetes	2014	Nyman et al. [72]	insulin stimulation: - steady state at different concentrations - time course response	primary human mature adipocytes: healthy and obese individuals with T2D
f	Decoupling of receptor and downstream signals in the Akt pathway by its low-pass filter characteristics	2010	Fujita et al. [74]	EGF (epidermal growth factor) time course	PC-12 cells (rat, pheochromocytoma)
g	Temporal Coding of Insulin Action through Multiplexing of the AKT Pathway	2012	Kubota et al. [75]	insulin time course	Fao cells (rat, hepatoma) primary rat hepatocytes (Wistar rat)
h	In Vivo Decoding Mechanisms of the Temporal Patterns of Blood Insulin by the Insulin-AKT Pathway in the Liver	2018	Kubota et al. [76]	hyperinsulinemic-euglycemic clamp conditions: insulin administration; glucose and somatostatin administration to suppress endogenous insulin secretion	male SD (Sprague Dawley) rats
i	Sensitivity control through attenuation of signal transfer efficiency by negative regulation of cellular signaling	2012	Toyoshima et al. [77]	EGF time course NGF (nerve growth factor) time course	PC-12 cells (rat, pheochromocytoma) HeLa cells (human, cervical cancer) Swiss 3T3 cells (mouse, embryonic fibroblasts) HUVEC cells (human, umbilical vein/vascular endothelium)
j	A Dynamic Network Model of mTOR Signaling Reveals TSC-Independent mTORC2 Regulation	2012	Dalle Pezze et al. [86]	insulin and amino acids time course	HeLa alpha Kyoto cells (human, cervical cancer) C2C12 cells (mouse, myoblasts)
k	Insulin Signaling in Insulin Resistance States and Cancer: A Modeling Analysis	2016	Bertuzzi et al. [101]	insulin, different concentrations, steady state in C2C12 cells treatment of L6 myotubes with medium enriched by proteins secreted by jejunal mucosa of non-diabetic mice versus medium enriched by proteins secreted by the mucosa of diabetic (db/db) mice	C2C12 cells (mouse, myoblasts) L6 cells (rat, myotubes)
l	A systems study reveals concurrent activation of AMPK and mTOR by amino acids	2016	Dalle Pezze et al. [108]	insulin and amino acids time course amino acids time course	C2C12 cells (mouse, myoblasts) HeLa alpha Kyoto cells (human, cervical cancer) MEF cells (mouse embryonic fibroblasts)
m	A modeling-experimental approach reveals insulin receptor substrate (IRS)-dependent regulation of adenosine monosphosphate-dependent kinase (AMPK) by insulin	2012	Sonntag et al. [113]	insulin and amino acids time course	HeLa alpha Kyoto (human, cervical cancer) C2C12 (mouse, myoblasts)
n	Dynamics of Elongation Factor 2 Kinase Regulation in Cortical Neurons in Response to Synaptic Activity	2015	Kenney et al. [114]	bicuculline time course	primary neuronal culture from P0 or P1 C57BL/6J mice
o	Systems-level feedbacks of NRF2 controlling autophagy upon oxidative stress response	2018	Kapuy et al. [115]	oxidative stress (data not shown)	human cells (not further specified)
p	Computational modeling of the regulation of Insulin signaling by oxidative stress	2013	Smith and Shanley [122]	*in silico* study	*in silico* study
q	The PI3K and MAPK/p38 pathways control stress granule assembly in a hierarchical manner	2019	Heberle et al. [123]	arsenite time course	MCF-7 cells (human, breast cancer) HeLa alpha Kyoto cells (human, cervical cancer) CAL51 cells (human, breast cancer) HEK293T cells (human embrionic kidney cells) LN18 cells (human, glioblastoma)

### Growth factor signaling to mTORC1

**Figure 2.**
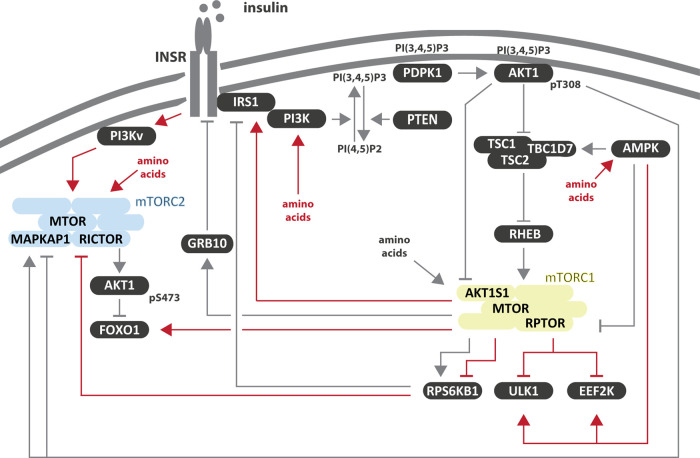
Growth factor (insulin) and nutrient (amino acids) signaling to the two MTOR complexes. Shown in red are edges described by computational studies in the last decade (Table 1).

Growth factors such as insulin are sensed by receptor tyrosine kinases. Upstream of mTORC1, the binding of insulin to the insulin receptor (INSR) results in the recruitment and tyrosine phosphorylation of the insulin receptor substrate 1 (IRS1) [24,43] (Figure 2). IRS1 is a scaffold for several proteins including the phosphoinositide 3-kinases (PI3K) [44]. The most prominent product of PI3K is phosphatidylinositol (3,4,5)-trisphosphate (PI(3,4,5)P3) [45]. PI(3,4,5)P3 can be metabolized by the inositol polyphoshphate-5-phosphatases INPP5D (inositol polyphosphate-5-phosphatase D) and INPPL1 (inositol polyphosphate phosphatase like 1) to phosphatidylinositol 3,4-bisphosphate (PI(3,4)P2) [46]. Both PI(3,4,5)P3 and PI(3,4)P2 promote the recruitment of proteins with a pleckstrin homology (PH) domain to the plasma membrane [46]. This includes the 3-phosphoinositide dependent protein kinase-1 (PDPK1) and AKT1 (reviewed by Hoxhaj and Manning [47]). The tumor suppressor PTEN (phosphatase and tensin homolog) functions as a PI3K antagonist to generate phosphatidylinositol 4,5-bisphosphate (PI(4,5)P2) and phosphatidylinositol 4-phosphate (PI(4)P) [48]. Upon PI3K activation and/or PTEN inactivation, PDPK1 is recruited to the plasma membrane and phosphorylates AKT1 at threonine 308 (AKT1–T308), thus leading to its activation [47]. AKT1 phosphorylates and inhibits the tuberous sclerosis (TSC) complex [49], as well as AKT1S1 [29,50], both negative regulators of mTORC1 [49,51–55]. The TSC complex comprises of TSC1 (Hamartin, TSC complex subunit 1), TSC2 (Tuberin, TSC complex subunit 2) and TBC1D7 (TBC1 domain family member 7) [56], and acts a GTPase activating protein (GAP) for the small GTPase RHEB (RAS homolog mTORC1 binding) [57–60]. When GTP bound, RHEB activates mTORC1 at the lysosomal surface [61]. mTORC1 phosphorylates a plethora of targets including RPS6KB1 (ribosomal protein S6 kinase B1) [62] and eIF4E-binding protein 1 (4E-BP1) [63] to promote biosynthetic processes and cellular growth.

mTORC1 activation by insulin is tightly balanced by several feedback loops. On the one hand, mTORC1 phosphorylates GRB10 (growth factor receptor-bound protein 10) [64,65], which in turn binds and inhibits the INSR. On the other hand, the mTORC1 substrate RPS6KB1 phosphorylates and inhibits IRS1 [66,67]. While biochemical studies identified the negative feedback loop from mTORC1/RPS6KB1 to the INSR/PI3K axis [64–67], computational studies added later a positive feedback loop from mTORC1 to IRS1 [68]. By measuring and simulating the mTORC1 response to insulin in adipocytes derived from healthy humans or type 2 diabetes (T2D) patients, Strålfors and colleagues used ODE-based modeling to investigate mechanisms of insulin resistance [68–73] (Table 1; a–e). Based on a series of modeling studies [68–72], they proposed that mTORC1 insensitivity towards insulin in T2D-derived adipocytes can only be simulated when assuming a positive feedback from mTORC1 to IRS1 (Figure 2). Upon T2D, attenuation of this positive feedback results in insulin insensitivity of the MTOR network. Whether this positive feedback translates to cellular systems other than adipocytes awaits further investigation. Also Kuroda and colleagues investigated in a series of modeling studies growth factor sensitivity of AKT1 and its targets *in vitro* and *in vivo* [74–77] (Table 1; f–i). They reported that distinct temporal patterns of growth factor signals to AKT1 (sustained versus pulsed) are selectively decoded by its downstream targets including mTORC1. While some AKT1 targets reflect a sustained response others reflect a pulsed response, allowing distinct functional outcomes to be mediated by the same pathway. Kubota et al. [75] also proposed an inhibitory input on RPS6KB1 downstream of AKT1 (Figure 2) leading to a signaling delay. It will be interesting to explore whether this mechanism involves RPS6KB1 targeting by the phosphatases PHLPP1/2 (PH domain and leucine rich repeat protein phosphatase 1/2) [78] and/ or PP2A (protein phosphatase 2 A) [79].

### Growth factor signaling to mTORC2

The signaling cascade activating mTORC2 upon growth factor stimulation (Figure 2) is currently under debate. Two studies proposed that mTORC2 activation by growth factors directly depends on PI3K-derived PI(3,4,5)P3 and PI(3,5)P2 [80,81]. Gan et al. [80] suggested that the mTORC2 component MAPKAP1 binds via its PH domain to PI(3,4,5)P3 at the plasma membrane. MAPKAP1-PI(3,4,5)P3 binding ablates an auto-inhibition and results in mTORC2 activation. Ebner et al. [81] found by live-cell imaging that mTORC2 activation only partially depends on PI3K, whereas another mTORC2 subpopulation at the plasma membrane is constitutively active. Also downstream of PI3K, the molecular mechanism regulating mTORC2 was discussed, with three modes of activation being proposed: (i) mTORC2 activation, downstream of PI3K/AKT1, directly depends on the TSC complex but is independent of the TSC complex’ GAP activity towards RHEB [82,83]; (ii) mTORC2 activation is indirectly regulated by the TSC complex, as its ablation induces an mTORC1-driven negative feedback on PI3K [84]; (iii) mTORC2 activation is independent of the TSC complex as mTORC2 enhances cell proliferation also in TSC2 knockout cells [85]. While it proved difficult to clarify the mode of mTORC2 activation by experiments only, data-driven ODE-based modeling [86] (Table 1; j) suggested that mTORC2 is neither directly nor indirectly activated by the TSC complex. Instead, mTORC2 is activated through a PI3K variant, which is independent of the negative feedback from mTORC1 (Figure 2). While insulin signaling to mTORC1 and 2 is separate at the level of PI3K, the two mTOR complexes are intertwined further downstream. RPS6KB1 downstream of mTORC1 phosphorylates RICTOR at threonine 1135 thus inhibiting mTORC2 [87–89]. Phosphorylation of MAPKAP1 at threonine 86 (MAPKAP1-T86) by AKT1 [90–92] and RPS6KB1 [90] has been proposed to alter mTORC2 activity, but it is unclear whether MAPKAP1-T86 phosphorylation is activating [91,92] or inhibitory [90]. In these studies, insulin dependent AKT1–pS473, downstream of mTORC2, was monitored while expressing mutagenized MAPKAP1-T86A. Whereas AKT1–pS473 was reduced after 10 min [91], it was enhanced after 30 min [90]. Thus, the discrepancy might come from measurements at different points of the signaling dynamic, and time course based computational modeling might be a suitable means to solve this issue. Another reason for the discrepancy might be the use of double [90] versus single [91] MAPKAP1 mutants, and thus also the interaction of different MAPKAP1 phosphorylation sites in mediating mTORC2-driven AKT1 phosphorylation dynamics might be worth investigating in future systems studies. While these approaches still await their realization, several computational studies have addressed the interconnection between mTORC1 and mTORC2. Magnusson et al. [71] (Table 1; d) dissected insulin-mediated mTORC1–mTORC2 crosstalk in the context of T2D. In adipocytes derived from T2D patients, mTORC2-mediated AKT1–pS473 was increased and mTORC1 activity was decreased as compared with adipocytes from non-diabetic humans. This behavior could be simulated by introducing a connection from RPS6KB1 to RICTOR that inhibits mTORC2, supporting the findings of several preceding experimental studies [87–89]. Also a possible connection between AKT1 and mTORC2 was addressed but could not be confirmed or refuted [71].

mTORC2 phosphorylates several AGC kinases including AKT1 [93], serum/glucocorticoid regulated kinase 1 (SGK1, [94]), and protein kinase C proteins (PRKCs; [95]). The activation of AGC kinases requires two phosphorylation events, one in the activation loop mediated by PDPK1 and the other in the hydrophobic motif, mediated by different kinases including mTORC2 (reviewed by Manning and Toker [96] and Pearce et al. [97]). The most widely used readout for mTORC2 activity is AKT1 phosphorylation at S473, but it has to be interpreted with caution as it can be influenced through conformational changes induced by phosphorylation at the activation loop [97]. Thus, the PDPK1 target site AKT1–T308 should be co-monitored to control for possible effects on the mTORC2 substrate site.

As AKT1 is targeted by mTORC2 and activates mTORC1, it is often proposed that mTORC2 is upstream of mTORC1 [22,40,96,98]. However, this hypothesis was challenged already early after mTORC2's discovery, as RICTOR knockout mice with abolished AKT1-S473 phosphorylation did not show changes in mTORC1 activity [99,100]. To the best of our knowledge, there is so far no evidence that mTORC2 activates mTORC1 via AKT1. This notion is also supported by a computational study [101] (Table 1; k), which dissected the regulation of mTORC1 by single (T308 or S473) or double (T308 and S473) phosphorylated AKT1 species and the relevance thereof in insulin resistance, cell cycle progression and cell death. Bertuzzi et al. [101] showed that single phosphorylation of AKT1–T308 is sufficient for full mTORC1 activation. Furthermore, AKT1–pS473 was detectable when PI3K was inactive and AKT1–T308 was dephosphorylated. This suggests that at least in some contexts, the two phosphorylation events are independent and determine substrate specificity rather than activity of AKT1 [33,99,102].

Further computational studies dissected forkhead box O1 (FOXO1) regulation by mTORC1 and mTORC2 in the context of insulin resistance in T2D [69,70] (Table 1; b,c). FOXO1 is an insulin-responsive transcription factor [103]. AKT1 — downstream of mTORC2 — phosphorylates and inhibits FOXO1, resulting in its rapid exclusion from the nucleus. In an experimental-computational approach, Rajan et al. [69,70] showed that reduced levels of AKT1-mediated FOXO1–S256 phosphorylation in T2D can be recapitulated by a model in which mTORC1 inhibition results in decreased FOXO1 translation. This finding was surprising as mTORC2 had been considered the main regulator of the AKT1-FOXO1 axis, and it suggests that in T2D signaling to FOXO1 shifts from mTORC2 to mTORC1.

### Amino acid signaling to MTOR

In response to amino acids, mTORC1 translocates to the surface of the lysosomes where it encounters its activator RHEB [59]. Hence, the lysosomal surface is considered as the main site of mTORC1 activation by amino acids (reviewed by Kim and Guan [40], and Liu and Sabatini [22]). The lysosomal translocation of mTORC1 is mediated by a complex machinery, which includes the RRAG GTPases (Ras-related GTP-binding) [104,105] and the Ragulator complex [61,106,107], a pentamer consisting of LAMTOR 1 to 5 (late endosomal/lysosomal adaptor, MAPK and MTOR activator 1 to 5) [106]. When active, the RRAG GTPases form heterodimers consisting of GTP-bound RRAGA or RRAGB with GDP-bound RRAGC or RRAGD [22,40]. Activation of the RRAG complexes involves different amino acid sensors [40]. Thus, lysosomal translocation is considered the main mTORC1 activating mechanism upon amino acid stimulation. However, a computational-experimental study which considered only one amino acid input directly impinging on mTORC1, thus mimicking mTORC1 lysosomal localization, could not recapitulate the amino acid-induced dynamics of the MTOR network [108]. Taking advantage of a combination of experimentation, ODE modeling, and text mining-enhanced quantitative proteomics, Dalle Pezze et al. [108] identified three additional amino acid inputs to the network, namely (i) mTORC2, (ii) PI3K, upstream of mTORC1, and (iii) AMP-activated protein kinase (AMPK) (Table 1; l, Figure 2). The latter observation was surprising as AMPK is canonically considered to be activated by nutrient deficiency and energy shortage (reviewed by Gonzalez et al. [109]). AMPK promotes catabolism (autophagy) by phosphorylating unc-51 like autophagy activating kinase 1 (ULK1) [110], and inhibits anabolism by phosphorylating TSC2 [111], and RPTOR [112]. Hence, AMPK and mTORC1 are typically considered as antagonists whose activity is mutually exclusive. However, four systems studies [108,113–115] (Table 1; l–o) showed that AMPK and mTORC1 are concomitantly activated. This discovery was probably due to the use of time-course data, as is typical for dynamic modeling studies, covering time points at which both kinases are active. Earlier, experimental studies relied on measurements at single or few time points, being the likely reason for missing concurrent AMPK and mTORC1 activity [116–118], highlighting the critical importance of the iterative combination of *in silico* network modeling with time series data to unravel signaling crosstalk. What is the biological importance of concomitant AMPK and mTORC1 activity? Dalle Pezze et al. [108] proposed that AMPK-driven catabolism is required to sustain the pools of intermediary metabolites for mTORC1-mediated anabolic processes. Kenney et al. [114] suggested that in neurons AMPK and mTORC1 converge on the eukaryotic elongation factor 2 kinase (EEF2K) to balance its activity and tightly control translation and synaptic function.

### Stress signaling to MTOR

**Figure 3.**
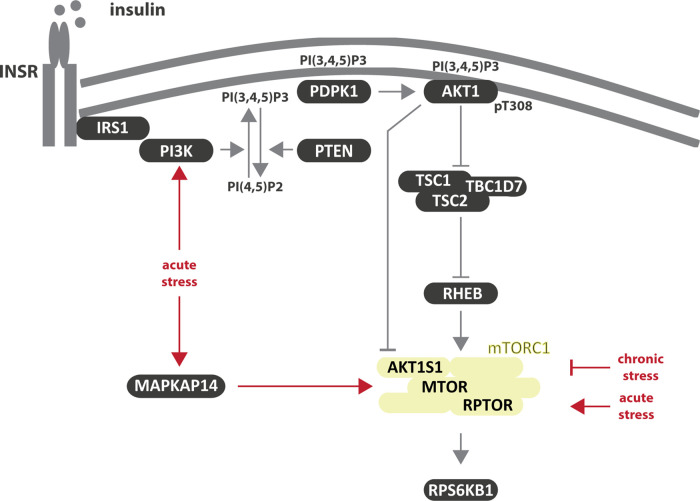
Stress signaling to mTORC1. Shown in red are edges described by computational studies (Table 1).

Next to metabolic signals, MTOR responds to numerous stressors including nutritional, oxidative, endoplasmic reticulum, and hypoxic stress [23,42]. The multitude of mechanisms transducing different stresses to mTORC1 have been reviewed by Heberle et al. [42]. Although stress inputs are often considered as inhibitory [42,119–121], also mechanisms activating mTORC1 have been reported (Figure 3). In an *in silico* analysis Smith and Shanley [122] suggested that chronic stress is inhibitory, while acute stress activates mTORC1 (Table 1; p). They analyzed these conditions with regard to insulin-induced dynamics on INSR, PI3K, AKT1 and FOXO1 and proposed that acute oxidative stress sensitizes the pathway to insulin while sustained oxidative stress results in the inhibition of the insulin response. Another computational-experimental study analyzed activating inputs on mTORC1 during acute stress upon sodium arsenite exposure [123] and identified PI3K and the MAP kinase p38 (MAPKAP14) as two major stress-responsive kinases that activate mTORC1 (Table 1; q, Figure 3). Dynamic modeling revealed a hierarchy between the two inputs, with PI3K being the pre-dominant mTORC1 activator and MAPKAP14 taking over when PI3K activity dropped.

## Conclusion

Systems studies have uncovered new crosstalk and mechanisms in the MTOR network. Thus, they complement experimental approaches and open new avenues to hypothesis building and testing in metabolic signaling. Next to applications in basic research, systems approaches are currently also being developed for medical applications [124,125]. Major funding initiatives for systems medicine are ongoing at both national and European level. The MTOR network is targeted directly and indirectly by many clinically approved small compounds [125]. Hence, patient specific and clinically validated MTOR network models might serve in the future to support therapy decisions for the treatment of cancer and other diseases [124,126] characterized by aberrant MTOR activity [22]. While some patents protect such applications for commercial use [127,128], they await their clinical validation. An important step in this direction will be the further development of criteria by the drug agencies to establish the credibility of computational tools for regulatory and clinical use [129].

## Perspectives

- Systems modeling complements experimental biology for hypothesis building and testing in metabolic signaling.
- Systems approaches constitute powerful tools to decipher complex network topologies and signaling dynamics upstream and downstream of MTOR.
- Computational models of metabolic signaling hold promise for applications in systems medicine.

## Open Access

Open access for this article was enabled by the participation of University of Groningen in an all-inclusive *Read & Publish* pilot with Portland Press and the Biochemical Society.

## Abbreviations

AKT1S1: AKT1 substrate 1
AMPK: AMP-activated protein kinase
DEPTOR: DEP domain containing MTOR interacting protein
EEF2K: eukaryotic elongation factor 2 kinase
EGF: epidermal growth factor
FOXO1: forkhead box O1
GAP: GTPase activating protein
GRB10: growth factor receptor-bound protein 10
INPPL1: inositol polyphosphate phosphatase like 1
G: INPP5D, inositol polyphosphate-5-phosphatase D
INSR: insulin to the insulin receptor
IRS1: insulin receptor substrate 1
LAMTOR 1 to 5: late endosomal/lysosomal adaptor, MAPK and MTOR activator 1 to 5
MAPKAP1: MAPK associated protein 1
MLST8: MTOR associated protein, LST8 homolog
MTOR: mechanistic target of rapamycin
mTORC1: mTOR complex 1
NGF: nerve growth factor
ODE: ordinary differential equation
PDPK1: 3-phosphoinositide dependent protein kinase-1
PI3K: phosphoinositide 3-kinases
PH: pleckstrin homology
PHLPP1/2: PH domain and leucine rich repreat protein phosphatase 1/2
PI(3,4,5)P3: phosphatidylinositol (3,4,5)-trisphosphate
PI(3,4)P2: phosphatidylinositol 3,4-bisphosphate
PI(4)P: phosphatidylinositol 4-phosphate
PI(4,5)P2: phosphatidylinositol 4,5-bisphosphate
PP2A: protein phosphatase 2A
PRKCs: protein kinase C proteins
PRR5/PRR5L: Proline rich 5/like
PTEN: phosphatase and tensin homolog
RHEB: RAS homolog mTORC1 binding
RICTOR: RPTOR independent companion of mTORC2
RPS6KB1: ribosomal protein S6 kinase B1
RPTOR: regulatory associated protein of mTORC1
RRAG: RAS-related GTP-binding
SGK1: serum/glucocorticoid regulated kinase 1
TBC1D7: TBC1 domain family member 7
TSC: tuberous sclerosis
TSC1: Hamartin, TSC complex subunit 1
TSC2: Tuberin, TSC complex subunit 2
TTI1/TELO2: TELO2 interacting protein 1/telomere maintenance 2
T2D: type 2 diabetes
ULK1: unc-51 like autophagy activating kinase 1
4E-BP1: eIF4E-binding protein 1
